# Syringomyelia: determining risk and protective factors in the conformation of the Cavalier King Charles Spaniel dog

**DOI:** 10.1186/2052-6687-1-9

**Published:** 2014-07-29

**Authors:** Thomas J Mitchell, Susan P Knowler, Henny van den Berg, Jane Sykes, Clare Rusbridge

**Affiliations:** School of Clinical Veterinary Science, University of Bristol, Langford, Bristol BS40 5DU UK; School of Veterinary Medicine, Faculty of Health & Medical Sciences, University of Surrey, Duke of Kent Building, Guildford, Surrey GU2 7TE UK; Fitzpatrick Referrals, Halfway Lane, Eashing, Godalming, Surrey GU7 2QQ UK; Lawson Imaging, Lawson Health Research Institute, 268 Grosvenor Street, London, Ontario N6A 4V2 Canada

**Keywords:** Syringomyelia, Chiari-like malformation, Chiari malformation, Cavalier King Charles spaniel, Conformation, Pedigree dog health, Canine skull morphology, Toy breed dogs, Occipital hypoplasia

## Abstract

**Background:**

Syringomyelia (SM) is a painful neurological condition, prevalent in brachycephalic toy breeds including the Cavalier King Charles Spaniel (CKCS). In these breeds, SM is typically secondary to Chiari-like Malformation (CM). There has been much debate in the scientific and veterinary communities to what extent head shape is indicative of either pathology, especially as certain craniosynostosis syndromes in humans (highly associated with CM) have characteristic facial and cranial morphologies. Elucidating a risk morphology would allow for selection away from these traits and proffer further breeding guidelines for the condition.

Dogs were measured in multiple countries by means of a standardised bony landmark measuring protocol and photo analysis by blinded, trained researchers.

**Results:**

The results found two significant risk factors in the conformation of the CKCS: extent of brachycephaly and distribution of cranium. The study identified a greater amount of cranium distributed caudally (relative to the amount distributed rostrally) to be significantly protective against syrinx development at the levels of three years of age, five years of age and when comparing a sample of SM clear individuals over the age of five to those affected younger than three years of age. A decreased cephalic index (decreasing brachycephaly) was significantly protective at the latter level. Cephalic index and caudal cranium distribution exhibited a negative, linear relationship. Cephalic index demonstrated a positive linear relationship with the amount of doming of the head.

**Conclusions:**

This study proposes a risk phenotype of brachycephaly with resulting rostrocaudal doming that is more rostrally distributed and hence sloping caudally.

The results of this study may allow for selection against risk aspects of conformation in the CKCS in combination with the British Veterinary Association/Kennel Club CM/SM scheme to enable reduction in CM/SM incidence. Further research comparing this external risk phenotype to the internal presentation upon MRI would determine how these features are indicative of syrinx development. Utilising breeds in which CM free individuals are more available may allow for validation of this risk phenotype for CM or determine alternatives.

## Lay summary

Syringomyelia (SM) is a painful condition, more common in toy breeds, including the Cavalier King Charles Spaniel (CKCS), than other breeds. In these toy breeds, SM is usually secondary to a specific malformation of the skull (called Chiari-like Malformation, CM for short).

There has been debate as to whether head shape is related to CM/SM, especially as some humans have similar characteristic facial and skull shapes, and what this may be. Identifying a head shape in dogs that is associated with these diseases would allow for selection away from these conditions and could be used to further breeding guidelines.

Dogs were measured in several countries using a standardised “bony landmark” measuring system and photo analysis by trained researchers.

This paper describes two significant risk factors associated with CM/SM in the skull shape of the CKCS: extent of brachycephaly (the broadness of the cranium (top of skull) relative to its length) and distribution of doming of the cranium.

The study showed that having a decreased cephalic index (less brachycephaly) was significantly protective. Further to this, more cranium at the back of the head (caudally) relative to the amount at the front of the head (rostrally) was significantly protective against disease development. This was shown at three and five years of age, and also when comparing a sample of “SM clear” individuals over five years to those affected under three years.

This study suggests that brachycephaly, with resulting rostrocaudal doming, is associated with CM/SM. These results could provide a way for selection against the risk head shape in the CKCS, and thus enable a reduction in CM/SM incidence. Studying other breeds in which CM free individuals are more frequent may validate this risk phenotype for CM too.

## Background

The Cavalier King Charles Spaniel (CKCS) is a toy breed dog, popular as a companion and also in the conformation showing fancy with 5,970 and 39,670 new registrations in 2012 with the Kennel Club (KC) and worldwide respectively [1]. The CKCS, like many brachycephalic toy breeds, is predisposed to syringomyelia, a condition where fluid filled cavities (syrinxes) develop within the central spinal cord. The resulting damage is associated with clinical signs of pain and variable neurological deficits, such as scoliosis and paresis [2].

Impedance of normal free flow of cerebrospinal fluid (CSF) through the foramen magnum appears to be a major factor responsible for the formation of a syrinx in the cervical spinal cord and in the CKCS. Syringomyelia (SM) is associated with Chiari-like Malformation (CM), a condition which is ubiquitous in the breed and also a cause of pain in some individuals [3–6]. CM is characterised by a mismatch between skull and brain volume and overcrowding of the craniocervical junction with compression of the CSF channels [5, 7]. Furthermore, SM has a prevalence of up to 70% in CM-affected CKCS, and it is unclear why some dogs develop SM and some do not [2, 8].

CM is analogous to Chiari type 1 and 0 malformation in humans. Some cases of Chiari type 1 malformations are associated with craniosynostosis, especially syndromic, multisuture, and lambdoid synostosis [9]. Some craniosynostosis syndromes are associated with classic facial and skull features, for example Crouzon’s syndrome, a disorder of the first branchial arch, which has an approximate 70% prevalence of Chiari I malformation. It is characterized by brachycephaly with exophthalmos, lateral strabismus and hypertelorism (greater than normal distance between the eyes). Furthermore, maxillary bone insufficiency results in psittichorhina (beak-like nose) and mandibular prognathism (undershot jaw) [10]. Previous studies in the Griffon Bruxellois have suggested that the shortening of the cranial base that characterizes CM may result in compensatory lengthening of other skull bones and that the radiographic appearance of the skull can be used to predict CM and SM [11, 12]. This suggests external conformational characteristics may relate to internal risk factors for SM development in the CKCS.

The clinical signs of CM/SM are pain (associated with either obstruction of CSF flow and/or neuropathic pain attributable to damage to the nervous tissue) and its behavioural indicators. Consequently, CM/SM is a highly debilitating and distressing disease for both dog and owner, highlighting the need for measures to reduce the prevalence of this condition within the population [13].

SM has been found to be progressive and late-onset in nature, making breeding decisions difficult without an indication of future disease status [2]. Currently, the British Veterinary Association (BVA) and KC Health Scheme for CM/SM requires brain and cranial cervical magnetic resonance imaging (MRI) of breeding dogs and subsequent selection of sire and dam to reduce the incidence in the progeny [1]. This scheme has been found to be effective until alternative tools become available [14]. However, due to the expense of such a procedure, requirement for anaesthesia and its late onset nature, additional tools that can identify individuals unsuitable for breeding from an earlier age would be beneficial. The aim of this study was to establish if head shape (by measuring) could proffer key indicators of risk of SM. We hypothesized that CM and the risk of SM may be associated with certain skull and facial characteristics in the CKCS. Furthermore, the risk conformation may reflect the characteristics seen in similar, Chiari-associated craniosynostosis syndromes of humans, secondary to an overall shortening of the skull base and specifically relating to cephalic index.

While understanding of conformational risk and protective factors for SM should not be used as a means of determining whether an individual should be bred or not (due to gene pool considerations), they could act as a useful selection pressure. This selection pressure could decrease the number of generations with which the prevalence of the condition is reduced, but also, by employing it at both the level of mate selection and offspring selection in breeding plans, could safeguard better results. It could also provide guidance to breed clubs, breeders and judges that have a duty to “avoid obvious conditions or exaggerations which would be detrimental in any way to the health, welfare or soundness of the breed” [15]. Furthermore, it may provide veterinarians with substantiated advice to provide to breeders outside the showing fancy and occasional hobbyists.

## Results

One hundred and thirty three CKCS were included in the study in the UK (n = 99), Canada (n = 31) and the Netherlands (n = 3). Thirty one CKCS were male. All conformational characteristics were normally-distributed (Table 1). Ages ranged from 1.5 to 13 years.

**Table 1:** **Summary of conformation of sample included in the study**

Characteristic	Mean (mm)	Standard deviation	95% Confidence interval (Lower - Upper)
Muzzle length	39.7	6.2	38.5 - 40.8
Height of lowest point of thorax	169.1	17.6	165.8 - 172.4
Height of tuber ischium	241.2	26.3	236.2 - 246.2
Body length	398.8	37.0	391.8 - 405.8
Palpebral aperture (medial to lateral canthus)	40.1	5.23	39.1 - 41.1
Eye separation relative to cranium length	2.1	0.2	2.0 - 2.1
Rostrocaudal doming	1.4	0.2	1.37 - 1.4
Lateral doming	2.2	0.2	2.1 - 2.2
Zygomatic arch depth from cranium	10.5	4.3	9.6 - 11.5
Cephalic index	108.2	12.4	105.9 - 110.6
Craniofacial index (typically referred to as cephalic index in dogs)	68.1	7.6	66.7 - 69.6

### Multivariable logistic analysis

Seventy nine individuals remained for the regression after removal of those SM clear under the age of three, and the distribution of disease status can be seen in Table 2. Univariate screening revealed variables to incorporate into each model for sequential removal (Table 3).

**Table 2:** **Disease status of sample included**

SM affected at any age	47
SM affected before 3 years of age	22
SM affected before 5 years of age	35
SM clear after 5 years of age	18

**Table 3:** **Results of univariate screening**

Dependent variable	Variables included in univariate screening
SM clear over three years of age (1) or affected younger than three (0)	Percentage of cranium in quadrant 4*, Palpebral aperture*, Eye separation relative to cranium length*, Cephalic index, Zygomatic arch-cranium width difference*, Eye conformational abnormality*, Rostrocaudal doming, Lateral doming, Craniofacial index
SM clear over five years of age (1) or affected younger than five (0)	Percentage of cranium in quadrant 4*, Palpebral aperture*, Eye separation relative to cranium length*, Cephalic index, Zygomatic arch-cranium width difference, Eye conformational abnormality, Rostrocaudal doming, Lateral doming, Craniofacial index
SM clear over five years of age (0) or affected younger than three years of age (1)	Percentage of cranium in quadrant 4*, Palpebral aperture*, Eye separation relative to cranium length*, Cephalic index*, Zygomatic arch-cranium width difference (placed in final model based on hypothesis), Eye conformational abnormality, Rostrocaudal doming, Lateral doming, Craniofacial index

*= p < 0.2.

### Syringomyelia clear over three years of age

**Figure 1:**
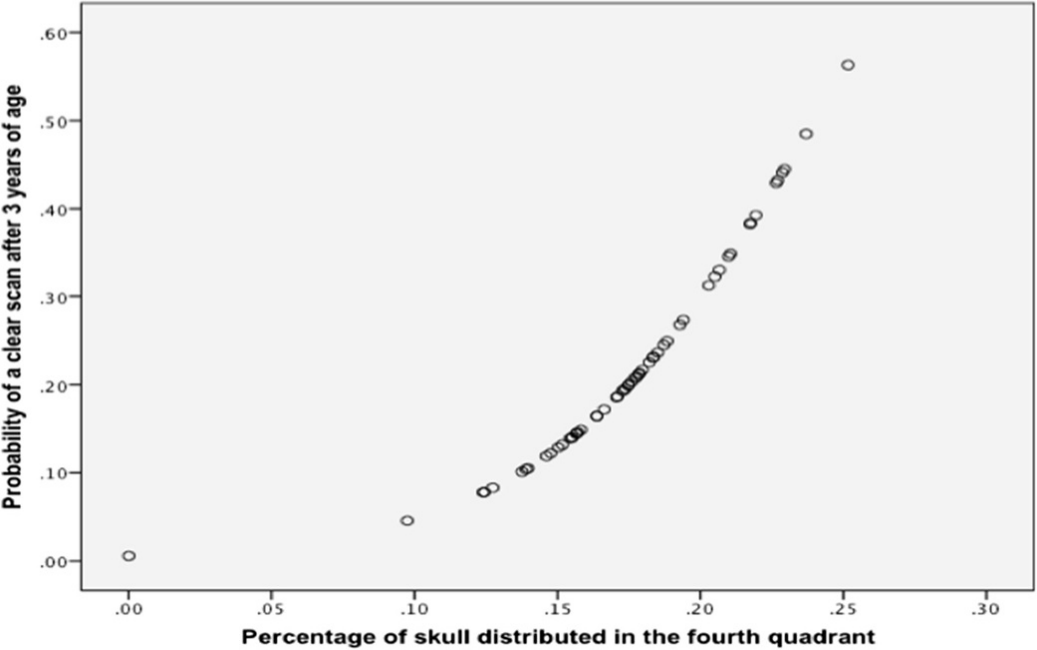
**MRI SM clear after the age of three and percentage of skull distributed in the fourth quadrant.** Graph illustrating relationship between percentage of skull distributed in the fourth quadrant upon photo analysis and the probability of that individual being clear over the age of three years. The graph shows risk of developing SM decreasing with increasing distribution of cranium caudally versus rostrally, suggesting dogs with significantly more rostral doming being more at-risk of developing SM before 3 years of age.

Direct logistic regression was performed on the independent variables that passed the univariate screening. The final model contained two independent variables (zygomatic arch-cranium width difference, percentage of cranium in quadrant four) and included 60 individuals (Table 4). The final model was statistically significant, χ^2^ (2, N = 60) = 12.8, p < 0.05, indicating that the model was able to distinguish SM affected and SM clear individuals over three years of age. The model as a whole explained between 19.2% (Cox and Snell R square) and 25.6% (Nagelkerke R square) of the variance in disease status. Furthermore, it was able to correctly classify 75% of cases into SM clear or affected with a specificity of 78.1% and a sensitivity of 71.4%. As seen in Table 4, one variable was significant and one represented a trend. The strongest predictor of being SM clear over the age of three, and therefore protective against the condition, was the percentage of the cranium in the fourth quadrant (p < 0.05) with an odds ratio of 1.9 × 10^9^, suggesting a very powerful predictor (Figure 1). This indicated that for every 1% of the cranium distributed in the fourth quadrant, cases were highly less likely to develop the condition before three years of age.

**Table 4:** **Multivariable logistic regression for SM clear over three years of age (1) versus not (0**)

Independent variable	Odds ratio	95% Confidence interval (Lower - Upper)	P value
Zygomatic arch-cranium width difference	1.1	1.0 - 1.3	0.126
Percentage of cranium distributed in fourth quadrant	1.9 × 10^9^	312.0 - 1.2 × 10^16^	0.007

### Syringomyelia clear over five years of age

**Figure 2:**
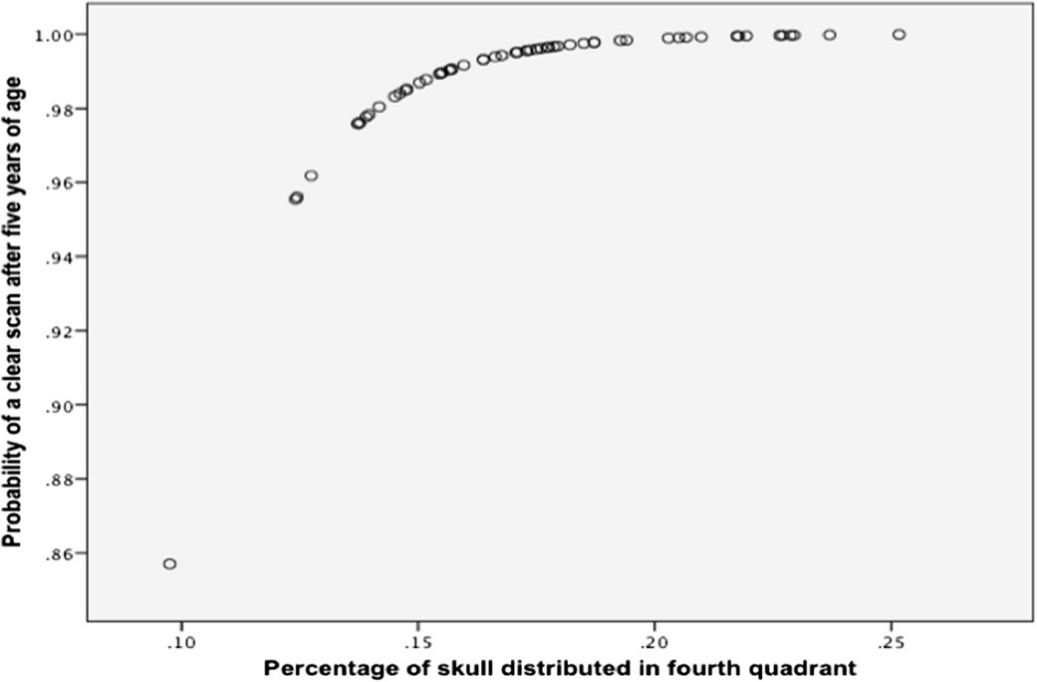
**MRI SM clear after the age of five and percentage of skull distributed in the fourth quadrant.** Graph illustrating relationship between percentage of skull distributed in the fourth quadrant upon photo analysis and the probability of that individual being clear over the age of five years. The graph shows increasing distribution of cranium caudally versus rostrally is significantly protective against developing SM before 5 years of age.

Direct logistic regression was performed on the independent variables that passed the univariate screening. The final model contained three independent variables (percentage of cranium in quadrant four, palpebral aperture and gender). Palpebral aperture was maintained based on R square values and gender was maintained for blocking purposes based on the predictive power of the model. The final model was statistically significant, χ^2^ (3, N = 65) = 28.6, p < 0.001, indicating that the model was able to distinguish SM affected and SM clear individuals over five years of age. The model as a whole explained between 36.5% (Cox and Snell R square) and 52.2% (Nagelkerke R square) of the variance in disease status. Moreover, it was able to correctly classify 88.9% with a specificity of 93.3% and a sensitivity of 77.8%. One variable was significant, one represented a trend and one was maintained for purposes of blocking (Table 5). The strongest predictor of being SM clear over the age of five, and therefore protective against the condition, was the percentage of the cranium in the fourth quadrant (p < 0.001) with an odds ratio of 6.8 × 10^20^, suggesting a very powerful predictor (Figure 2). This indicated that for every 1% of the cranium distributed in the fourth quadrant, cases were highly less likely to develop the condition before five years of age.

**Table 5:** **Multivariable logistic regression for SM clear over five years of age (1) versus not (0)**

Independent variable	Odds ratio	95% Confidence interval (Lower - Upper)	P Value
Percentage of cranium distributed in fourth quadrant	6.8 × 10^20^	2.2 × 10^9^ - 2.1 × 10^32^	< 0.001
Palpebral aperture	0.9	0.7 - 1.0	0.099
Gender	0.4	0.1 - 1.9	0.237

### Syringomyelia clear over five years of age versus syringomyelia affected younger than three years of age

Cephalic index and percentage of skull in the fourth quadrant (photo analysis), a significant association (p < 0.05) was found. However, these variables did share measurements; therefore, two separate models were created to avoid losing information. Similarly a trend was found between zygomatic arch-cranium width difference and palpebral aperture, likely indicative of a limit of palpebral aperture based on the width of the head itself; therefore, these were separated also.

**Figure 3:**
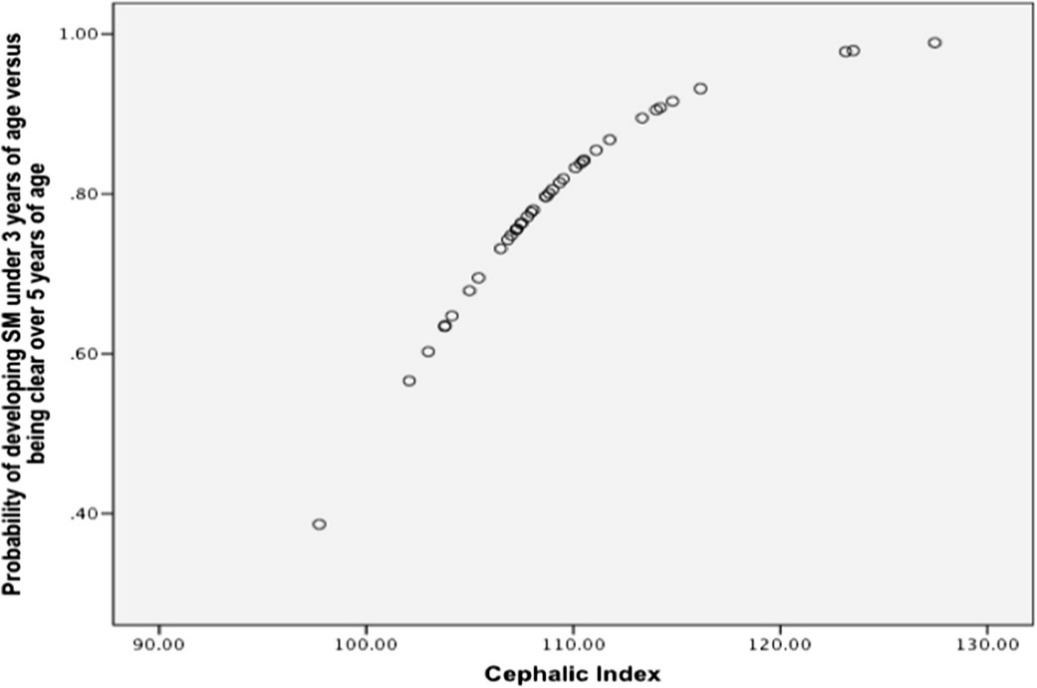
**MRI SM affected younger than three versus SM clear over five and cephalic index.** Graph illustrating relationship between cephalic index and the probability of that individual being SM affected under the age of three versus SM clear over the age of five. This figure shows increasing cephalic index to be a significant risk factor in developing SM before 3 years of age when comparing to dogs that are clear over 5 years of age.

Direct logistic regression was performed on the independent variables that passed the univariate screening. The final model contained two independent variables (cephalic index and zygomatic arch-cranium width difference). Zygomatic arch-cranium width difference was maintained in the model based on observations of R square. The final model was statistically significant, χ^2^ (2, N = 39) = 7.3, p < 0.05, indicating that the model was able to distinguish SM clear over the age of five individuals to those SM affected younger than three. The model as a whole explained between 17% (Cox and Snell R square) and 22.6% (Nagelkerke R square) of the variance in disease status. Moreover, it was able to correctly classify 69.2% with a specificity of 63.2% and a sensitivity of 75%. One variable in the final model was significant (Table 6). This variable was cephalic index with an odds ratio of 1.2, a risk factor for SM. This indicated that for every one unit increase in cephalic index, there was a 1.2 increase in risk of developing SM before the age of three compared to being clear over five (Figure 3).

**Table 6:** **Multivariable logistic regression for SM affected younger than three (1) versus clear over five (0)**

Independent variable	Odds ratio	95% Confidence interval (Lower - Upper)	P Value
Cephalic index	1.2	1.0 - 1.4	0.026
Zygomatic arch-cranium width difference	0.9	0.7 - 1.1	0.169

A separate model including palpebral aperture and percentage distributed in fourth quadrant, as in the second model, for SM clear over five versus affected younger than 3 was found to be significant (Table 7). Furthermore, it revealed a similar descriptive value with 31.1% (Cox and Snell R square) and 41.5% (Nagelkerke R square) explanation of the variance in disease status. Odds ratios for percentage of cranium distributed in fourth quadrant approached zero due to it being strongly protective against early disease development.

**Figure 4:**
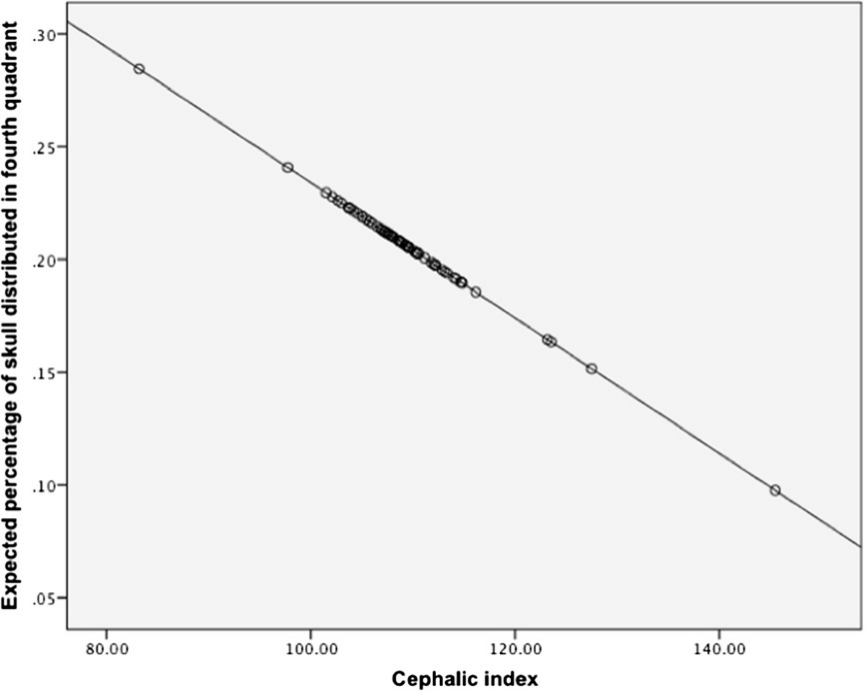
**Percentage of skull distributed in the fourth quadrant and cephalic index.** Graph illustrating the linear relationship determined between cephalic index and percentage of skull distributed in fourth quadrant upon photo analysis. This figure demonstrates that increasing cephalic index in this breed is associated with the doming of the cranium distributing more rostrally (rather than caudally) in a linear fashion. This suggests why both of these features of breed conformation are risk factors.

Further analysis revealed a significant (p < 0.05) linear, negative relationship between cephalic index and percentage of skull distributed in fourth quadrant (Percentage in fourth quadrant = 0.5 - 0.003 × Cephalic index; Figure 4). A significant (p < 0.001) relationship between cephalic index and the amount of doming in the rostrocaudal direction was found (rostrocaudal doming = 0.3 + 0.011 × Cephalic index).

**Table 7:** **Multivariable logistic regression for SM affected younger than three (1) versus clear over five (0)**

Independent variable	Odds ratio	95% Confidence interval (Lower - Upper)	P Value
Percentage of cranium distributed in fourth quadrant	8.8 × 10^-18^	1.6 × 10^-29^ - 4.9 × 10^-6^	0.004
Palpebral aperture	1.2	1.0 - 1.4	0.134

## Discussion

### Conformational indicators

The study found two aspects of conformation to be associated with the development of SM in the CKCS: the cephalic index and the distribution of cranium across the length of the head. It was found that a higher cephalic index and, separately, a lower percentage of the cranium distributed caudally were significantly associated with disease development.

**Figure 5:**
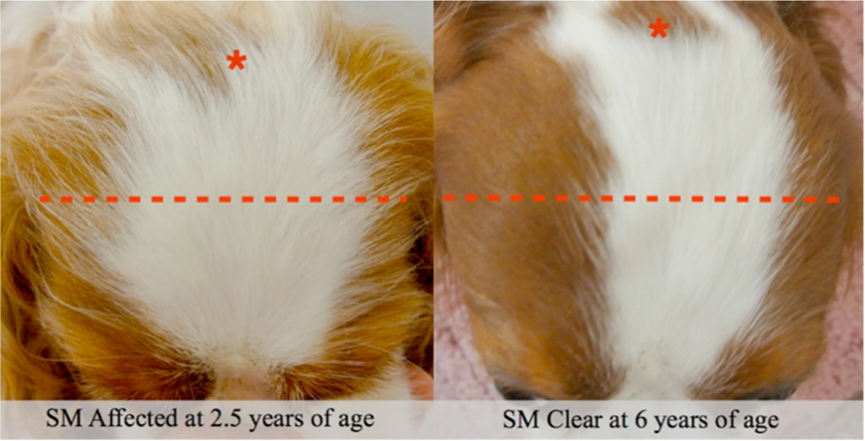
**Example of cephalic index differences in young affected and older clear individuals.** The shorter and broader cranium is clearly demonstrated in the early SM affected individual. Red asterisk represents the point of the occipital protuberance and the line represents the breadth of the head at its widest points.

The human medical definition of cephalic index was used in this study, and the findings indicate that as the cranium is shortened and broadened, the risk of developing SM increases (Figure 5). In this case, the indicator is specifically at the level of below three years compared to those clear over five years of age, indicating it was protective against developing the condition at a young age but also protective in maintaining SM clear status over the age of five. Limited variability in this trait across the sample, seen in the narrow confidence interval upon exploration, may explain the limited variation in condition presence across the population. Craniofacial index was not a significant indicator, demonstrating the unimportance of muzzle length in disease progression. The increasing cephalic index in individuals was found to be highly associated with a more domed head.

**Figure 6:**
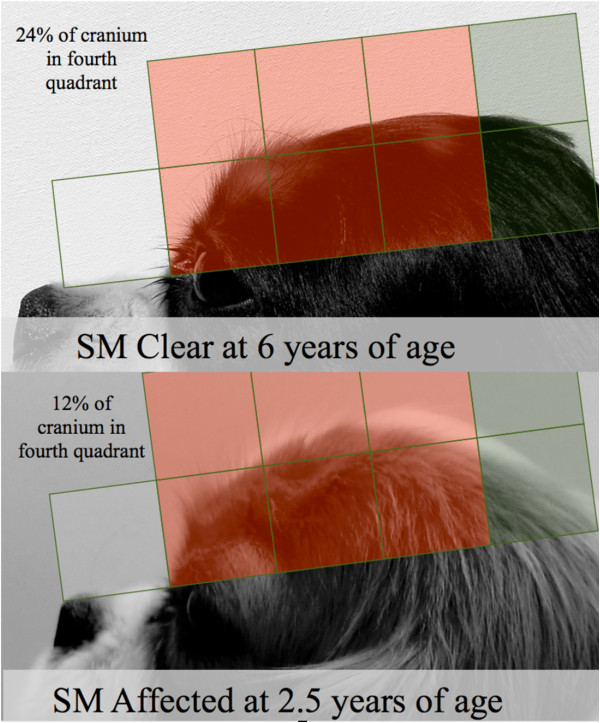
**Example of caudal cranium distribution differences in young affected and older clear individuals.** The cranium in the fourth quadrant in the SM clear individual shows an increased coverage of the grid relative to the first, second and third quadrants, resulting in a high percentage distribution in the fourth quadrant of 24%. Conversely, in the early SM affected individual, an increased coverage in the first, second and third quadrants and a decreased coverage in the fourth quadrant leads to a decreased percentage cranium distribution in the fourth quadrant of 12%.

A higher percentage of cranium distributed in the fourth quadrant of the head was found to be significantly protective at all age levels analysed (most caudal area; green, Figure 6). As this was a percentage, the final value depended on two qualities: the amount of cranium in the fourth quadrant (green, Figure 6) but also the amount of cranium distributed rostrally to that quadrant (red, Figure 6). This inclusion of two qualities of head shape in the variable may explain the very high odds ratios.

Further analysis of dependence revealed a strong association between the two characteristics. The two variables did not share information. An explanation for this association may be a limit of conformation in nature or they may represent components of a common type within the breed. Based on plausible selection pressures, the loss of cranium distributed towards the back of the head could be a consequence of selection for a broader and shorter head (a higher cephalic index) and/or selection for more doming towards the front of the head to produce a deeper stop. No significant gender differences were apparent in these traits.

The results that were not significant but maintained in the final model may have been found to be significant (or not) with more accurate divisions in phenotype or a larger population. Of particular interest is palpebral aperture. It is a misconception that the “large” eye stipulated in the breed standard of the CKCS reflects the size of the globe; instead it reflects the aperture and degree of globe exposure. The trends relating to palpebral aperture found in two separate models both suggest that an increasing palpebral aperture is indicative of disease presence; however, the breed standard directly requires the breed to have “large” eyes [16]. Further characterisation of the relationships between brachycephaly and other aspects of cranial morphology, as well as contributing to the evidence-base on other associated conditions (such as airway obstruction), would proffer further conformational indicators and a more complete illustration of risk phenotype.

These findings are in parallel to those of the radiographic study performed on the Griffon Bruxellois that found that shortening of the basicranium was associated with CM, a rounding of the skull dorsally (compensatory parietal lengthening) and a comparatively broad head [11]. A further MRI study on CM and SM affected Griffon Bruxellois found increased height of the rostral cranial cavity was the most significant predictor of disease [12]. Previous observations of the changes in CKCS skull morphology included concavity, or flattening of the supraoccipital bone and caudal cranium.

As the foetus develops, the cartilaginous foundation of the base of the skull is replaced with bone by the process of endochondral ossification. Cranial synchondroses, however, remain within the neonate and represent cartilaginous boundaries where growth can continue through continual production of cartilaginous matrix and ossification. Later, complete ossification of the plates determines the end of bone growth. The closing of these synchondroses has been found to differ between bones but also between breeds of dog [17, 18]. This study supports the hypothesis of an overly short skull base through premature synchondrosis closure - a craniosynostosis - in the CKCS, resulting in a shortening of the basicranial axis and compensatory lengthening of other bones, especially of the calvaria. The craniosynostosis, as well as reducing the rostrocaudal length of the skull and contributing to neural tissue overcrowding may also reduce the volume of the jugular foramen, which may cause an intracranial hypertension and predispose syrinx development. Schmidt et al. (2013) determined a craniosynostosis relative to mesaticephalic breeds (for example, Labrador and German shepherd dog) in the CKCS spheno-occipital synchondrosis. This synchondrosis contributes especially to post-natal cranial base elongation in humans, and the difference in closure time found in the CKCS was also significant when comparing to other brachycephalic breed such as Pugs and Pekingese [19]. The study findings of loss of cranium distributed towards the back of the head could also support a CM hypothesis of occipital hypoplasia [8].

The conformational indicators found were able to correctly classify between 69% and 89% of cases as SM clear or affected at various age levels. Furthermore, they were found to explain up to 50% of the variability in condition presence. This suggests that these morphological characteristics of the skull are implicated in, or at least indicative of, the pathogenesis of SM as discussed above. This study was unable to determine conformational indicators at the level of CM in the CKCS due to lack of CM clear controls; however, the age at which an individual develops a syrinx may relate to slight graduations in CM so may be an indirect measure of CM severity or type.

A cephalic index calculation (Table 3; Figure 5) may represent a particularly useful measure in that it can be calculated with little skill (with low-cost tools), is visually obvious and gives a numerical output. The authors chose not to estimate a cut-off value for cephalic index as the information would be better employed in decreasing the cephalic index with each generation in the form of a selection pressure rather than removing individuals outright from the limited gene pool. This numerical output would be particularly effective in a population where trait variability was low, as demonstrated here, and could be incorporated into Estimated Breeding Value calculations or as part of the Mate Select system of the Kennel Club in a similar manner to that of hip scores.

## Conclusions

The conformational indicator of caudal cranium distribution was found to significantly, correctly classify cases as SM clear or affected at the level of three years of age, five years of age, and when comparing a sample of SM clear dogs over five years to those affected and younger than three. Cephalic index was able to significantly, correctly classify cases at the latter level. Results suggest that these indicators are irrelevant of age (after 18 months of age), gender and parity. These, therefore, represent invaluable tools in determining breeding plans in that they are not only protective against developing the condition in the first three years of life but they are protective against developing the condition at all, maintaining SM clear status beyond the age of five years.

Anecdotally, many breeders believe that selection for a larger, broader head decreases SM risk; however, selection for a broad head in any toy breed may in fact increase the risk of CM and SM, highlighting the need for widespread provision of guidance. The findings of this study allow for guidance to breeders and judges and appropriate selection of champions (likely to contribute morphologically to the population more than other individuals). This guidance could also be supplied to veterinarians who are an obvious point-of-contact to those breeders outside the showing fancy.

## Methods

### Population

Participants were recruited in multiple countries (Canada, Netherlands, U.K.) through a social media group, breed clubs, by word of mouth and as participants of previous studies. Owner consent for the non-invasive data collection process was gained from each participant. To participate, owners had to have one or more dogs that had undergone a brain and cervical MRI scan and have had it interpreted by a diplomate neurologist or radiologist. MRI scans were performed prior to the study as part of the breeding scheme or for diagnostic purposes. MRI reports had to be linked to dog by microchip to ensure that each dog’s phenotype recorded corresponded to the correct MRI report.

Participants were required to send these MRI reports to one of the authors (SPK), independent of all those performing measuring, and, after consistency checks, each dog was assigned a unique identification number, which was linked to microchip for purposes of blinding, and disease status recorded. Prior to analysis, this unique identification number was used to link each dog’s measurements to its information from the MRI report.

One hundred and thirty three CKCS were included in the study. An instruction manual of measurements was created by the authors. The majority of the dogs were measured by TJM (n = 82) who remained blinded to the MRI status until all the measurements were obtained. Additionally, some dogs were measured by researchers trained to take the measurements. Each individual was required to perform a pilot measurements study, which was assessed by TJM who would then ensure that the technique was modified if necessary.

All individuals included in the study had been diagnosed with CM. Individuals determined as clear of SM upon MRI scanning and under the age of 3 were removed from the sample due to the late-onset nature of the condition [1, 2]. These, however, were maintained up to the point of logistic regression for blinding purposes and to assess the homogeneity within the population. Dogs were categorised into three groups for analysis: SM affected younger than 3 years of age; SM clear over three years of age; SM clear over five years. These were chosen in accordance with the BVA & KC CM/SM breeding scheme age groups [1]. There were symptomatic dogs in each group, including those SM clear but affected by CM.

### Data collection

**Figure 7:**
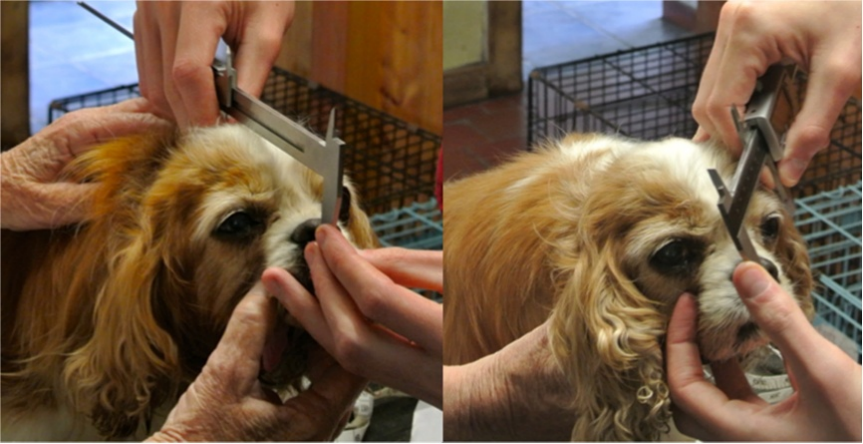
**Examples of measurements using bony and static landmarks.**

Dogs were measured according to a catalogue of standardised, piloted measurements, using bony or static landmarks. Measurements were designed to precisely and consistently characterise the conformation of the head (Table 8; Figure 7). Measurements of the body were also taken for purposes of providing proportions. Callipers, tape measure and ruler were used.

**Figure 8:**
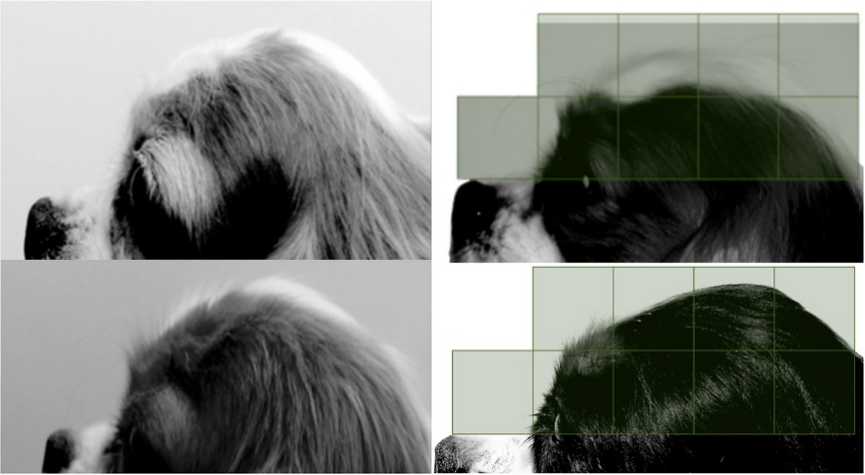
**Examples of photographs using the criteria for later analysis and of the way photos were scaled against the grid for analysis.** Each square represents a quadrant of 100 1 mm by 1 mm squares. Where the cranium extended beyond the first line, counting continued into the square above.

A photo of each dog was taken in accordance with criteria (Figure 8). The criteria were that the photo was taken against a white background with the head centred, parallel to the ground and with neck extended. For analysis, a standard grid made up of four quadrants, each containing 100 1 mm by 1 mm squares, was placed over photos displayed on a tablet device. Photos were rescaled such that the lower limit of the grid was aligned with the topline of the muzzle, the lateral border of the first quadrant was aligned with the most rostral point of the cranium and the lateral border of the last, or fourth, quadrant encompassed the remainder, most caudal aspect, of the cranium (Figure 8). Squares containing cranium were counted for each quadrant, a total determined, and, from this, the number from each quadrant was divided by the total number across all four quadrants to produce a percentage distribution of cranium per quadrant. Grid placement, scaling and analysis is inevitably subjective; therefore, to minimise individual differences in this method, the above was performed by a single, blinded analyst (TJM), and, to minimise differences in photo position, only within-photo ratios were used in the analysis (as opposed to absolute dimensions).

**Table 8:** **Summary of measurements included in the study with landmarks**

Measurement (mm)	Tool	Point A	Point B
M1	Callipers	Occipital protuberance	Medial nose-mouth transition
M2	Callipers	Occipital protuberance	Stop (point of transition from cranial to facial skeleton)
M3	Tape measure	Occipital protuberance	Stop
M4	Ruler	Stop	End of muzzle
M5	Tape measure	Most rostral point caudal to zygomatic arch on one side	Most rostral point caudal to zygomatic arch on the other side
M6	Callipers	Most rostral point caudal to zygomatic arch on one side	Most rostral point caudal to zygomatic arch on the other side
M7	Callipers	Zygomatic arch on one side	Zygomatic arch on the other side (the widest point)
M8	Callipers	Occipital protuberance	Point of wing of atlas
M9	Callipers	Lateral canthus of one eye	Lateral canthus of the other eye
M10	Callipers	Medial canthus of one eye	Medial canthus of the other eye
M11	Tape measure	Most ventral point of thorax	Ground
M12	Tape measure	Tuber ischium of the pelvis	Ground
M13	Tape measure	Greater tubercle of the humerus	Tuber ischium of the pelvis
M14	N/A	Eye conformational abnormalities, including strabismus, exophthalmos and exotropia determined by observation when dog looking forwards, left and right (Figure 9).

### Data analysis

Analysis of the data was performed using SPSS version 19 for Macintosh (©SPSS Inc.).

**Figure 9:**
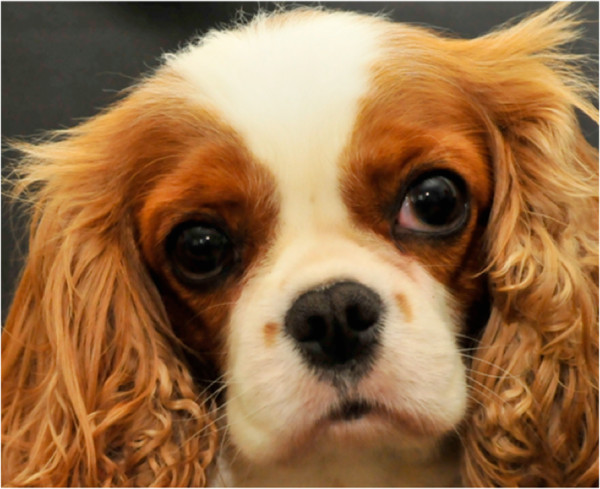
**Example of an eye conformational abnormality (left exotropia) in a Cavalier King Charles Spaniel.** Eye conformation abnormalities recorded in the study included strabismus, exopthalmos and exotropia in the study.

**Figure 10:**
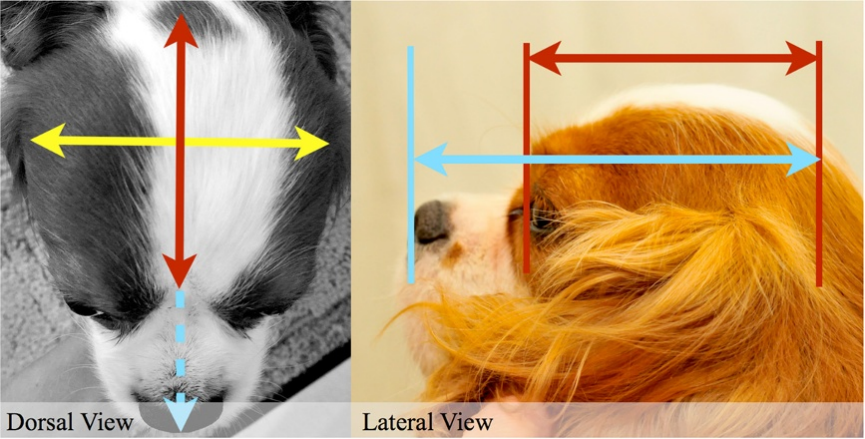
**Cephalic and craniofacial indices.** Cephalic index and craniofacial index were calculated as described in Table 9 using the calliper measurements illustrated here.

All independent variables (after transformations) were continuous except for eye conformational abnormality presence (M14; Figure 9), which was coded in a binary fashion. Measurements were combined to best quantify head morphology (Table 9; Figure 10).

**Table 9:** **Summary of transformations performed prior to analysis**

Variable name	Transformation
Palpebral aperture	Measurement between lateral canthi (M9) minus that of medial canthi of eyes (M10).
Eye separation relative to cranium length	Cranial length (M2) divided by distance between medial canthus of each eye (M10).
Rostrocaudal doming	Length across the top of the cranium (M3) divided by length of cranium (M2)
Lateral doming	Width across the top of the cranium directly behind the zygomatic arches (M5) divided by the width of the cranium at that point (M6).
Zygomatic arch deviation from cranium	Width over both zygomatic arches at the widest point (M7) minus the width of the cranium directly caudal to zygomatic arches (M6). This is then divided by the width over the zygomatic arches (M7).
Cephalic index	The width of the cranium (M6) (Figure 10; yellow) divided by its length (M2) (Figure 10; red).
Craniofacial index (typically referred to as cephalic index in dogs)	The width of the cranium (M6) (Figure 10; yellow) divided by length of cranium and muzzle (M1) (Figure 10; blue).

In order to determine the degree of homogeneity within the population, descriptives on various aspects of conformation were calculated.

A backwards-stepwise logistic regression approach was utilised for three dependent variables: (1) SM clear over three years of age or affected younger; (2) SM clear over five years of age or affected younger; (3) SM affected under the age of three or SM clear over the age of five. The sample itself and sample size varied with each analysis based on availability of dogs in the population (Table 10).

**Table 10:** **Number of individuals included in each model**

Dependent variable	Sample size
“SM clear over three years of age” or affected younger than three	60
“SM clear over five years of age” or affected younger than five	65
“SM clear over five years of age” or affected younger than three years of age	39

Univariate screening was performed based on the hypothesis of classic skull features seen in humans and a skull base craniosynostosis and those with a significance of less than 0.2 were built into the model and removed sequentially. Where an association between independent variables was found, the strongest predictor was used in the model.

## Glossary

**Brachycephaly** describes a developmentally normal type of skull with a high cephalic index, such as in snub-nosed breeds of dog such pugs and bulldogs.

The cephalic index is the ratio of the width of the cranium of an organism (taken behind the cheekbones in this study) divided by its length (i.e., in the horizontal plane, or front to back). It is usually expressed as a %. It differs from craniofacial index in that it does not relate to the length of the muzzle.

**Caudally:** towards the back of the head.

**Rostrally:** towards the front of the head.

**Cranium**: top part of the skull.

**Rostrocaudal:** doming that is more rostrally distributed and hence sloping caudally.

## Abbreviations

CKCS: Cavalier King Charles spaniel
CM: Chiari-like malformation
SM: Syringomyelia
CSF: Cerebrospinal fluid
KC: Kennel club
BVA: British veterinary association.
